# Supplementary figures and images for: Opposing actions of co-released GABA and neurotensin on the activity of preoptic neurons and on body temperature
Source: eLife. 2024 Aug 29;13:RP98677. doi: 10.7554/eLife.98677 (PMC11361704; doi:10.7554/eLife.98677)

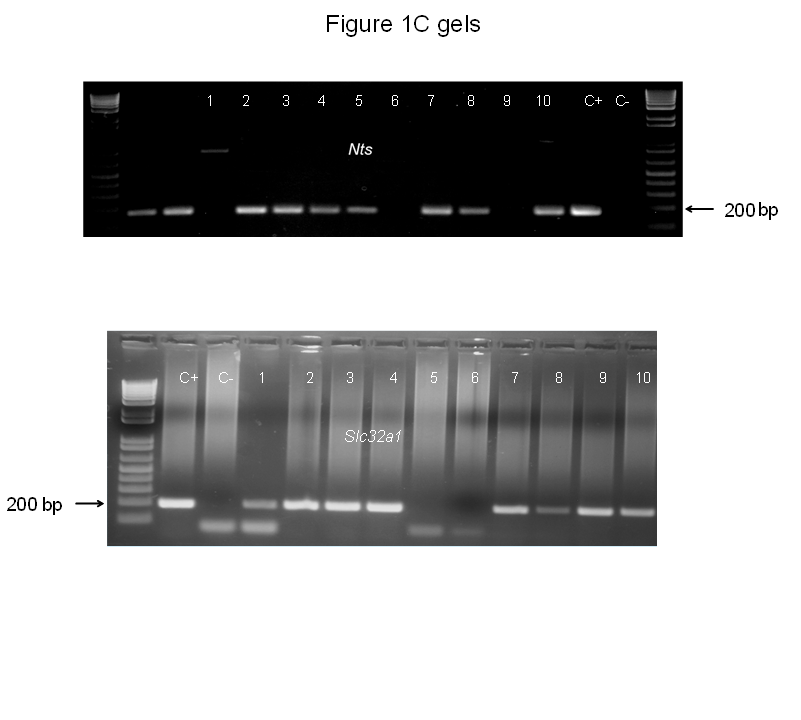

Supplement: Figure 1—source data 2. [file elife-98677-fig1-data2.zip › Figure 1- source data files/Figure 1- source data3.tif]

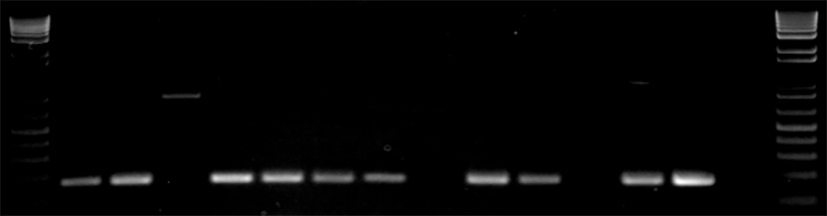

Supplement: Figure 1—source data 2. [file elife-98677-fig1-data2.zip › Figure 1- source data files/Figure 1-Nts-source data1.tif]

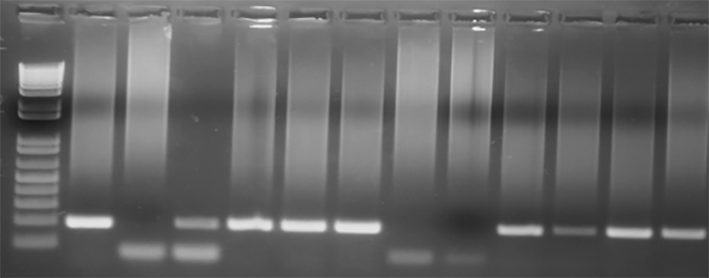

Supplement: Figure 1—source data 2. [file elife-98677-fig1-data2.zip › Figure 1- source data files/Figure 1-Slc32a1-source data2.tif]

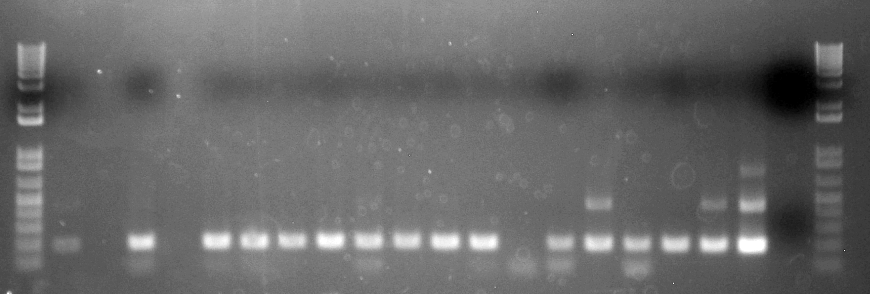

Supplement: Figure 2—source data 2. [file elife-98677-fig2-data2.zip › Figure 2- source data files/Figure2C-source data1.tif]

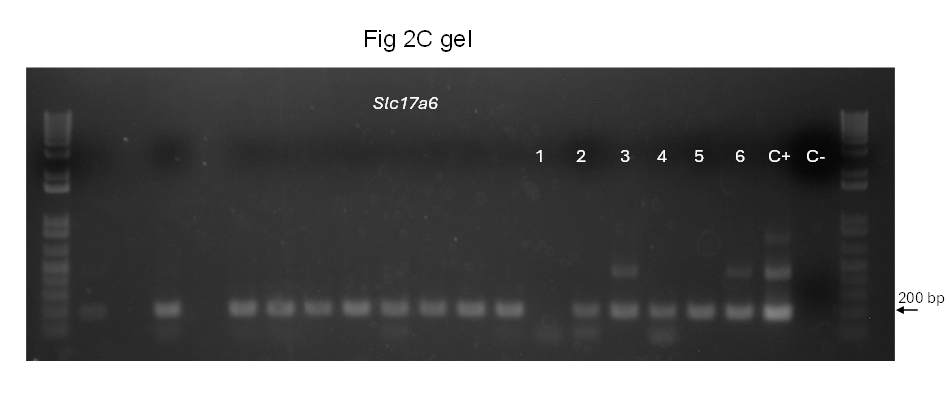

Supplement: Figure 2—source data 2. [file elife-98677-fig2-data2.zip › Figure 2- source data files/Figure2C-source data2.tif]
