# Supplementary material for: Opposing actions of co-released GABA and neurotensin on the activity of preoptic neurons and on body temperature
Source: eLife. 2024 Aug 29;13:RP98677. doi: 10.7554/eLife.98677 (PMC11361704; doi:10.7554/eLife.98677)
Supplement: Supplementary file 1. [file elife-98677-supp1.docx]

**Supplementary Table 1. PCR primers for the genes studied**

|  | Gene | External Primers | Ampli-con size | Internal Primers | Ampli-con size |
| --- | --- | --- | --- | --- | --- |
| 1 | *Neurotensin (Nts)* | F: AGGCCCTACATTCTCAAGAG  R: CATTGTTCTGCTTTGGGTTA | Bp:  398 | F: GGGGTTCCTACTACTACTGA  R: CATCACATCCAATAAAGCAC | Bp:  149 |
| 2 | *Slc32a1 (VGAT)* | F:GTCACGACAAACCCAAAGATCAC  R**:GTTGTTCCCTCATCATCTTCGCC** | Bp:  137 |  |  |
| 3 | *Adcyap1*  *(PACAP)* | F: CCTACCGCAAAGTCTTGGAC  R:TTGACAGCCATTTGTTTTCG | Bp:  181 |  |  |
| 4 | *Slc17a6*  *(Vglut2)* | F:  CTGGATGGTCGTCAGTATTTTATG  R:  ATGAGAGTAGCCAACAACCAGAAG | Bp:  503 | F:GCAGGAGCTGGACTTTTTATTTAC  R:TAGTTGTTGAGAGAATTTGCTTGC | Bp:  186 |

**Supplementary Table 2. P-values for Tukey’s test comparisons among groups (Fig 2B)**

| Pair | P-value |
| --- | --- |
| x_1_-x_2_ | 0.0001476 |
| x_1_-x_3_ | 0.0003445 |
| x_1_-x_4_ | 0.0005521 |
| x_1_-x_5_ | 0.007513 |
| x_1_-x_6_ | 0.01494 |
| x_2_-x_3_ | 1.676e-8 |
| x_2_-x_4_ | 1.751e-7 |
| x_2_-x_5_ | 1.178e-8 |
| x_2_-x_6_ | 1.447e-7 |
| x_3_-x_4_ | 0.006143 |
| x_3_-x_5_ | 0.0007085 |
| x_3_-x_6_ | 0.002418 |
| x_4_-x_5_ | 0.0003605 |
| x_4_-x_6_ | 0.000312 |
| x_5_-x_6_ | 0.2656 |

**Supplementary Table 3. P values of the** **Tukey’s test comparisons among groups (Fig 5B)**

| Columns | P-value |
| --- | --- |
| x1-x2 | 7.1x10^-5^ |
| x1-x3 | 0.7237 |
| x2-x3 | 7.82x10^-6^ |

**Supplementary Table 4. P values of the Tukey’s test comparisons among groups (Fig 7F)**

| Pair | P-value |
| --- | --- |
| x1-x2 | 0.044 |
| x1-x3 | 0.4212 |
| x1-x4 | 0.0201 |
| x2-x3 | 0.543 |
| x2-x4 | 0.978 |
| x3-x4 | 0.3303 |

**Supplementary Table** **5. P values of the Tukey’s test comparisons among groups (Fig 7G)**

| Pair | P-value |
| --- | --- |
| x1-x2 | 0.0001877 |
| x1-x3 | 0.05677 |
| x1-x4 | 0.001055 |
| x2-x3 | 0.04938 |
| x2-x4 | 0.8145 |
| x3-x4 | 0.2338 |

**Supplementary Table 6. P values of the Tukey’s test comparisons among groups (Fig 7H)**

| Pair | P-value |
| --- | --- |
| x1-x2 | 0.003388 |
| x1-x3 | 0.01139 |
| x1-x4 | 0.04768 |
| x2-x3 | 0.9318 |
| x2-x4 | 0.5657 |
| x3-x4 | 0.8872 |

**Supplementary Table 7.** **P values of the Tukey’s test comparisons among groups (Fig 7I)**

| Pair | P-value |
| --- | --- |
| x1-x2 | 0.001157 |
| x1-x3 | 0.00516 |
| x1-x4 | 0.2825 |
| x2-x3 | 0.8803 |
| x2-x4 | 0.04888 |
| x3-x4 | 0.1844 |
